# Nrf2 is required to maintain the self-renewal of glioma stem cells

**DOI:** 10.1186/1471-2407-13-380

**Published:** 2013-08-10

**Authors:** Jianhong Zhu, Handong Wang, Qing Sun, Xiangjun Ji, Lin Zhu, Zixiang Cong, Yuan Zhou, Huandong Liu, Mengliang Zhou

**Affiliations:** 1 Medical School of Nanjing University, No. 22, Hankou Road, Nanjing, Jiangsu 210089, China; 2 Department of Neurosurgery in Jinling Hospital, Neurosurgical Institution of People’s Liberation Army of China, No. 305, East Zhongshan Road, Nanjing, Jiangsu 210002, China; 3 Neurosurgery Department of Southern Medical University, No. 1838, Guangzhou Avenue, Guangzhou, Guangdong 510515, China

## Abstract

**Background:**

Glioblastomas are deadly cancers that display a functional cellular hierarchy maintained by self-renewing glioma stem cells (GSCs). Self-renewal is a complex biological process necessary for maintaining the glioma stem cells. Nuclear factor rythroid 2-related factor 2(Nrf2) plays a significant role in protecting cells from endogenous and exogenous stresses. Nrf2 is a key nuclear transcription factor that regulates antioxidant response element (ARE)-containing genes. Previous studies have demonstrated the significant role of Nrf2 in the proliferation of glioblastoma, and in their resistance to radioactive therapies. We examined the effect of knocking down Nrf2 in GSCs.

**Methods:**

Nrf2 expression was down-regulated by shRNA transinfected with lentivirus. Expression levels of Nestin, Nrf2, BMI-1, Sox2 and Cyclin E were assessed by western blotting, quantitative polymerase chain reaction (qPCR) and immunohistochemistry analysis. The capacity for self-renewal *in vitro* was assessed by genesis of colonies. The capacity for self-renewal *in vivo* was analyzed by tumor genesis of xenografts in nude mice.

**Results:**

Knockdown of Nrf2 inhibited the proliferation of GSCs, and significantly reduced the expression of BMI-1, Sox2 and CyclinE. Knocking down of Nrf2 changed the cell cycle distribution of GSCs by causing an uncharacteristic increase in the proportion of cells in the G2 phase and a decrease in the proportion of cells in the S phase of the cell cycle.

**Conclusions:**

Nrf2 is required to maintain the self-renewal of GSCs, and its down-regulation can attenuate the self-renewal of GSCs significantly.

## Background

Glioblastoma multiforme (GBM) is a lethal brain tumor. The median survival is approximately 14 months, even with aggressive surgery, radi0- and chemotherapy [1,2]. Recent studies have shown that some cells in gliomas retain many features of neuronal progenitor cells, including the ability to grow as neurospheres in culture, self-renew, and migrate in the brain [3-5]. These cells retain features of neural stem cells (NSCs), and we have referred to these particular cells as glioma stem cells (GSCs). They express the NSCs surface markers CD133 and Nestin [6-9]. There are novel opportunities for developing therapeutics by targeting the differentiation and self-renewal features of glioma. Unfortunately, GSCs are often resistant to either radio- or chemotherapy [10-12]. Although these cells represent only a small fraction of the tumor bulk, their high self-renewal capacity is thought to sustain tumor growth. The signaling pathways that maintain the proliferative capacity of these cells offers great potential for a better understanding of tumor genesis and development.

Nuclear erythroid-2-related factor 2 (Nrf2) is a redox-sensitive, basic leucine zipper protein that regulates the transcription of several antioxidant genes. It is a key nuclear transcription factor that regulates antioxidant response element (ARE)-containing genes [13,14]. The factor regulate gene include GSH synthesis, glutathione reductase and peroxidase families, NAD(P)H: quinone oxidoreductase1 (NQO1) [13]. Recent studies have shown multi-regulating potentials in many steps of cell biology [15,16]. The anti-tumor effects of Nrf2 were found to be mediated by its regulatory roles during glioma cell differentiation and growth inhibition *in vitro*[17,18]. However, the role of Nrf2 cell signaling pathway during self-renewal of GSCs is unclear. We hypothesize Nrf2 influences the proliferation of GSCs, which induce the relapse and invasion of glioblastoma.

In this study, we examined the role of Nrf2 in GSCs-by knocking down of Nrf2 with short hairpin RNAs (shRNAs) and decreased the proportion of spheres of GSCs. The cell cycle distribution of GSCs also changed with the variation of Nrf2 expressional level. Sox2, BMI-1 and Cyclin E had been identified playing an important role in self-renewal of GSCs. In our study, we found these biomarkers down-regulated by knocking down of Nrf2, which infer the relationships between Nrf2 and self-renewal. Developing of xenografts in node mice confirm repression of proliferation when the transcription level of Nrf2 decreased by shRNA. Finally, Nrf2 depletion was found to block the proliferation of human glioma both in vitro and *in vivo*.

## Methods

### Experimental procedures

Our study design was approved by the Ethics Committee of Jinling Hospital (Nanzi20120017). Patients that were recruited to our study provided written informed consent for participation to permit the scientific using their samples. Our animal experiments were approved by the Animal Ethics Committee of the Animal Experiment center at Jinling Hospital (SCXK 2012-012).

### Cell culture and treatment

Primary human glioblastoma G1, G2 and G3 cells were derived from freshly resected human surgical glioblastoma specimens. These were obtained from three patients of the Department of Neurosurgery in Jinling Hospital (Nanjing, P.R. China) and grown as tumor- spheres as former reported [19-21]. All samples were identified glioblastoma (WHO IV) by pathologists of Jinling Hospital. Tumors were dissociated with 0.25% trypsase and released by gentle pipetting and filtrated through a 70 μm cell strainer. Adherent culture of cells were performed by plating the cells in a gelatin-coated plastic flask in DMEM for 24 h and washed with PBS to remove red blood cells and cell debris. The tumor cells were collected and seeded in neural stem cell (NSC) medium (Gibco, USA) which combined Knockout medium with Neural-supplement, human recombinant basic fibroblast growth factor (bFGF, 50 ng/mL, Gibco, USA), epidermal growth factor (EGF, 50 ng/mL, Gibco, USA), penicillin (100 units/mL, Sigma, USA), streptomycin (100 ug/mL, Sigma, USA), and L-glutamine (2 mmol/L, Sigma, USA), and the density of 200 cells/cm2 to obtain floating tumor-spheres. Primary GSCs were incubated at 37°C in a atmosphere containing 5% carbon dioxide for 5 to 7 days. The medium was half-renewed every 3 days. Once cells were greater than 100 um in diameter, they were tentatively defined as GSCs spheres.

### Preparation of the lentivirus

Lentiviruses vectors for expression of Scrambled or Nrf2 shRNA was diluted in NSC medium containing 6 ug/ml polybrene. The shRNAs were then added to GSC cultures after 72 hours, transfected cells were selected using puromycin (5 ug/ml) for 24 hours. The human Nrf2 shRNA sequence was 5′-GCAGTTCAATGAAGCTCAACT-3′, while the scrambled shRNA sequence was 5′-TTCTCCGAACGTGTCACGT-3′. The lentivirus vectors were purchased from GenePharma Co., Ltd. (Shanghai, China).

### Secondary sphere propagation and sphere formation assay

To evaluate effects of knocking down of Nrf2 on GSCs population, primary GSCs were non-infected (control group) and infected with lentivirus for expression of either Scrambled or Nrf2 shRNA and selected for puromycin resistance as above. Following infection, the transduced cells were seeded into 24-well plates. After 72 hours, cells were dissociated with Accutase (Sigma-Aldrich, USA) for 15 min and re-seeded into culture dishes (100 mm in diameter) at a density of around 200 cells/cm2. At 96 hours after re-seeding, the number of sphere-like colonies was assessed by two independent scorers who were unaware of the sample designation.

For comparing spheres formation efficiency of colonies, GSCs were transduced with lentiviral particles as described above. At 72 hours after transduction, cells were dissociated with Accutase (Sigma-Aldrich, USA) and seeded at a density of 200 cells/cm2 in 24 well plates in quadruplicate, using culture medium supplemented with 10% fetal bovine serum (FBS). Cells were re-feed every 2 days, and after 2 weeks, cells were stained by gentian violet. The number of neuronal sphere-like colonies and differentiated colonies with a diameter greater than 50 um was counted by two independent scorers.

### Western blotting

Total protein lysates were prepared using an immunoprecipitation cell disruption and nuclear protein preparation kit (Beyotime, China). The cell disruption kit was supplemented with phenylmethanesulfonyl fluoride (PMSF), a protease inhibitor, and protein concentrations determined with a Bradford Protein Assay Kit (Beyotime, China). Nuclear proteins were separated using sodium dodecyl sulfate polyacrylamide gel electrophoresis (SDS PAGE) on 8–12% gradient gels. Separated proteins were transferred onto PVDF membranes (Millipore, Germany), the membrane was cut into narrow piece according to the protein molecular massive marker (Therme, USA), blocked with 5% non-fat milk for 1 hour at room temperature and probed with the appropriate antibody. Primary and secondary antibodies were diluted in 3% (w/v) bovine serum albumin (BSA) and secondary antibodies diluted in Tris-buffered saline (TBS) supplemented with 0.1% Tween 20 (TBST), respectively. Membranes were incubated with primary antibodies overnight, at 4°C, and with secondary antibody for 60 minutes, at room temperature. Following incubation with primary and secondary antibodies, membranes were washed three times (10 minutes per wash) with TBST, and developed by incubating in enhanced chemiluminescence(ECL)substrate (Millipore, Germany) for 5 minutes at room temperature. The fluorescent signal was detected with black-white films (Kodak, USA). The primary antibodies used were against the following proteins: Nrf2 (1:1000 dilution, Abcam, UK), Cyclin E (1:1000 dilution, Abcam, UK), Sox2 (1:1000 dilution, Epitomic, UK), BMI-1 (1:500 dilution, Epitomic, UK), and Histone H3 (1:500 dilution, Abcam, UK). The secondary antibody was an anti-rabbit-IgG conjugated to horseradish peroxidase (HrP) (Bioworld, USA) and was used at a 1:5,000 dilution.

### RNA isolation and qPCR

The GSCs were infected or infected with lentiviruses vectors expressing either Scrambled or Nrf2 shRNA, as described above. At 72 post-transduction, RNA was isolated from three independent cell culture preparations, and cDNA was synthesized using a Strand cDNA Synthesis Kit (Takara, Japan). Levels of transcripts for specific genes were determined by SYBR Green qRT-PCR, using gene specific primers for human transcripts encoding Nrf2, NQO1, HO-1, Sox2, BMI-1, Cyclin E, Nestin, GFAP and GAPDH. Primary sequences provided in supplementary material Table 1.

**Table 1:** Sequences of primers for remaining human genes

**Gene**	**Primer sequence**
Nrf2	5′TCAGCGACGGAAAGAGTATGA3′(F)
	5′CCACTGGTTTCTGACTGGATGT3′(R)
GAPDH	5′GAAATCCCATCACCATCTTC3′(F)
	5′CCACTGGTTTCTGACTGGATGT3′(R)
BMI-1	5′ TCTAAGGAGGAGGTGAA 3′(F)
	5′ TCTAAGGAGGAGGTGAA 3′(R)
Sox2	5′ CCCCTGTGGTTACCTCTTCCT 3′(F)
	5′ CCGTTAATGGCCGTGCC 3′(R)
Cyclin E	5′ ACCAGTTTGCGTATGTGA 3′(F)
	5′ TGTGGGTCTGTATGTTGTG 3′(R)
GFAP	5′ AGGGACAATCTGGCACAGG 3′(F)
	5′ CGGTAGTCGTTGGCTTCG 3′(R)
HO-1	5′TCTCCGATGGGTCCTTACACTC3′(F)
	5′GGCATAAAGCCCTACAGCAACT3′(R)
NQO1	5′ATGGTCGGCAGAAGAGC3′(F)
	5′GGAAATGATGGGATTGAAGT3′(R)

### Cell cycle analysis by flow cytometry

GSCs were seeded at a density of 1 × 10^6^ cells per 100 mm plate. After 24 hours, cells were infected with Scrambled or Human Nrf2 shRNA lentiviral constructs, followed by puromycin selection, as described above. At 72 hours post-infection, cells were prepared for cell cycle analysis by using propidium iodide (PI) staining and a Cell Cycle and Apoptosis Analysis Kit (Beyotime, China), according to the manufacturer’s protocol. Floating cells were included in the GSCs cell cycle analysis. Flow analyses were performed by the UNMC Cell Analysis core facility.

### Immunocytochemistry

GSCs infected with Scrambled or Nrf2 shRNA lentiviruses were seeded into a 24-well plates, previously coated with 0.1% gelatin or Matrigel, respectively. At 24 hours post-seeding, wells were washed three times with 500 ul of PBS. Cells were then fixed with 4% formaldehyde (Sigma-Aldrich, USA) in PBS for 20 minutes at room temperature. Cells were washed twice with PBS, permeabilized with PBS containing 0.1% Triton X-100 in PBS for 10 minutes at room temperature, and washed three times in PBS, before blocking. Cells were stained for Nrf2, Sox2, BMI-1, Cyclin E, Nestin and GFAP and blocked using 5% BSA(Sigma-Aldrich, USA) in PBS for 1 hour at room temperature. Following blocking, cells were incubated with Nrf2, Sox2, BMI-1, Cyclin E mixed with an anti-Nestin primary antibody overnight, at 4°C on a rocking platform. Cells were washed three times in PBS and the appropriate secondary antibody added, then allowed to incubate for 1 hour in the dark at room temperature. Cells were then washed three times in PBS, stained with DAPI and washed twice in PBS. Cells were photographed with a fluorescence microscope (Cral Ziess, Germany). We used primary antibodies against: Nrf2 (1:100 dilution, rabbit polyclone, Abcam, UK), Sox2 (1:200 dilution, rabbit monoclonal, Abcam, UK), Cyclin E(1:200 dilution, rabbit monoclonal, Abcam, UK), BMI-1 (1:100 dilution, rabbit monoclonal, Abcam, UK), Nestin (1:100 dilution, biobyte, mouse polyclone, UK). Secondary antibodies were against mouse-IgG conjugated to FITC (Sigma-Aldrich, USA), and an anti-rabbit-IgG conjugated to Cy3 (F0382, Sigma-Aldrich, USA). Nuclei were visualized using DAPI (Sigma-Aldrich, USA) at 1:10,000 in PBS.

### Xenografts

GSCs were maintained in serum-free NSCs medium containing Scrambled or Nrf2 shRNA lentiviruses for 3 days. Cell viability was determined using trypan blue staining. Viable cells (2 × 10^4^) were dissociated in 50 μl of PBS and implanted subcutaneously into the flanks of male nude mice (4 weeks old, *n* = 6 mice per group) that were randomly selected (4 weeks old, *n* = 6 mice per group) that were randomly selected and divided into three groups. After 5 weeks, animals were sacrificed and xenograft tumors measured and subjected to immunofluorescent analysis. Tumor growth was measured every week and tumor volume calculated as (length/2) × width^2^. All animal experimental protocols used in this study were in accordance with Institutional Guidelines for Animal Experiments and nude mice were maintained at The Center for Experimental Animals of Nanjing University.

### Statistical analysis

Results from all experiments were presented from at least three independent replicates. We used SPSS10.0 statistical software to analyze results by Student’s *t*-test or ANOVA as applicable. Values are presented as the mean ± standard deviation (SD).

## Results

### Nrf2 knockdown disrupts self-renewal and pluripotency of GSCs

**Figure 1:**
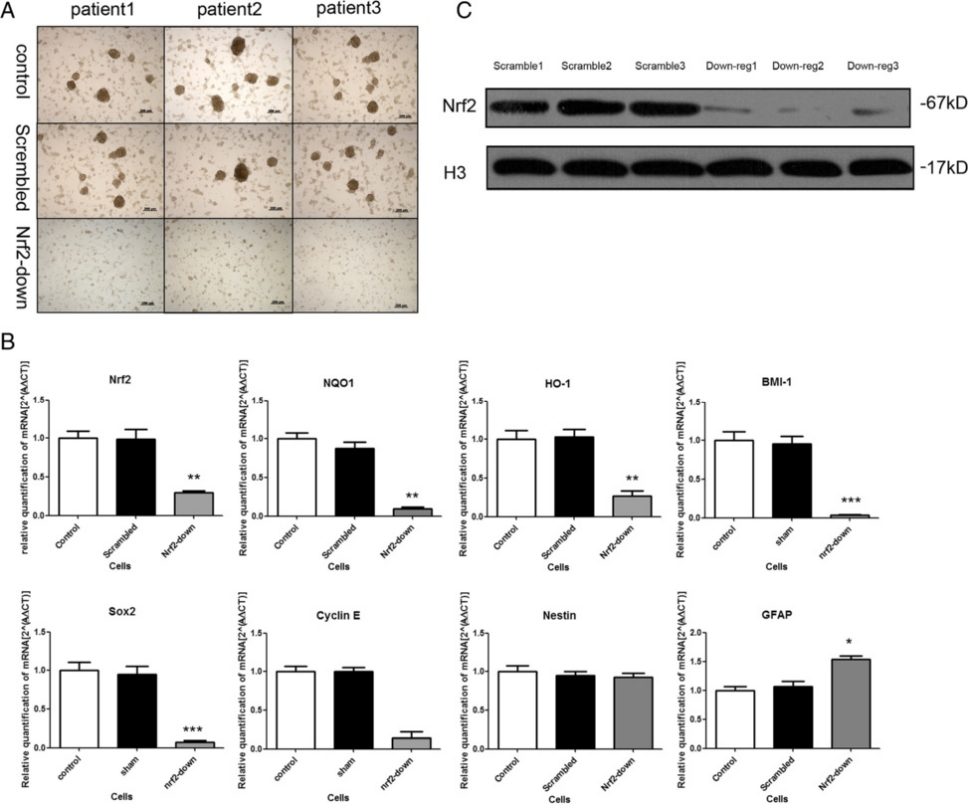
**Knockdown of Nrf2 in GSCs causes changes in cell morphology, elevation of gene markers of proliferation and reduction in the protein levels of Nrf2. (A)** Photomicrographs of representative GSC colonies infected with lentiviral constructs for Scrambled shRNA and control group, neural spheres of Nrf2-downregulated group were smaller than other two groups. **(B)** qRT-PCR of RNA transcripts isolated from GSCs after transduction. BMI-1, Sox2, Cyclin E, NQO-1and HO-1 decreased with the knocking down of Nrf2. GFAP increased and Nestin not changed obviously. **(C)** Western blot assay of the Nrf2. *: P < 0.05; **: P < 0.01; ***: P < 0.005.

**Figure 2:**
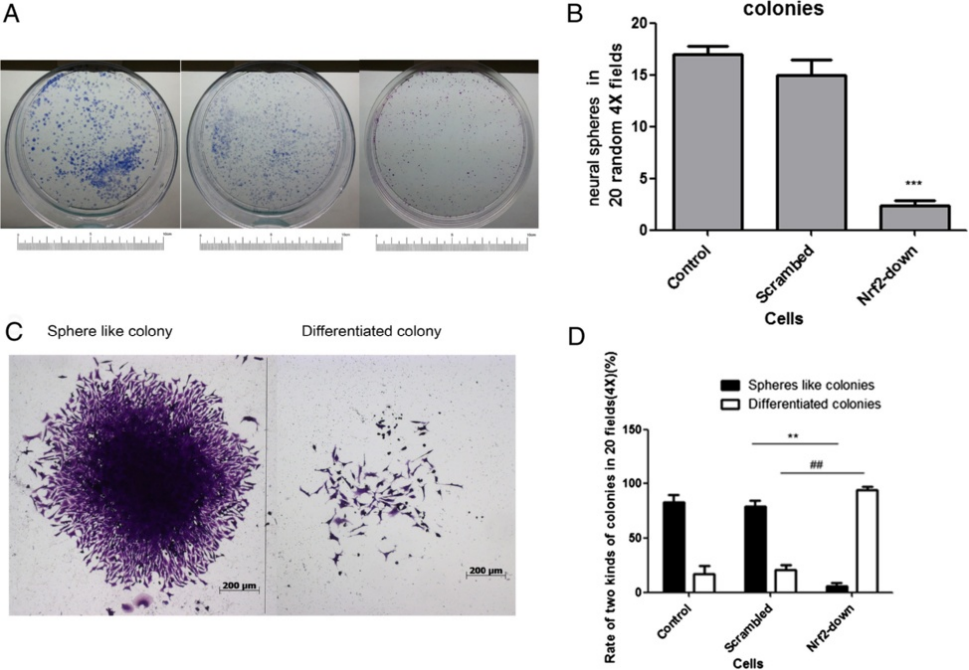
**Self-renewal and the phenotype of GSCs are disrupted upon Nrf2 knockdown. (A)** Photo of cloning efficiency of GSCs transduced with either Scrambled or Nrf2 shRNA lentiviral constructs. **(B)** gentian violet positive colonies in 20 random 4X field. **(C)** Representative photomicrographs of sphere-like (left) and mixed-differentiated (right) colonies of cells. **(D)** Quantification of sphere-like and mixed-differentiated-like colonies. The proportion of spheres like colonies is lower in Nrf2-downregulated group.

To determine how the knockdown of Nrf2 affected the fate of GSCs, two independent observers unaware of sample designation counted colonies from 20 randomly selected low-power fields (4× magnification) 96 h after cells were subcultured (Figure 1A). For both sphere densities, knockdown of Nrf2 reduced self-renewal of GSCs 3-fold compared with control cells (Figure 2A and 2B). Following knockdown of Nrf2 levels in GSCs using multiple shRNA constructs, we observed that cells lost their characteristic phenotype and differentiated (Figure 2C and 2D). In GSC cultures infected with the Scrambled shRNA, there was an 82.67 ± 7.37% increase in the number of sphere-like colonies, whereas this was 5.67 ± 3.06% when Nrf2 was knocked down with Human Nrf2 shRNA (Figure 2D). There was a significant decrease in the number of sphere-like colonies (*P* = 0.0047) and a significant increase in differentiated-like colonies (*P* = 0.0033) upon Nrf2 knockdown.

### Subcellular localization of pluripotency-associated markers and the Nrf2-asso- ciated protein after knockdown of Nrf2

**Figure 3:**
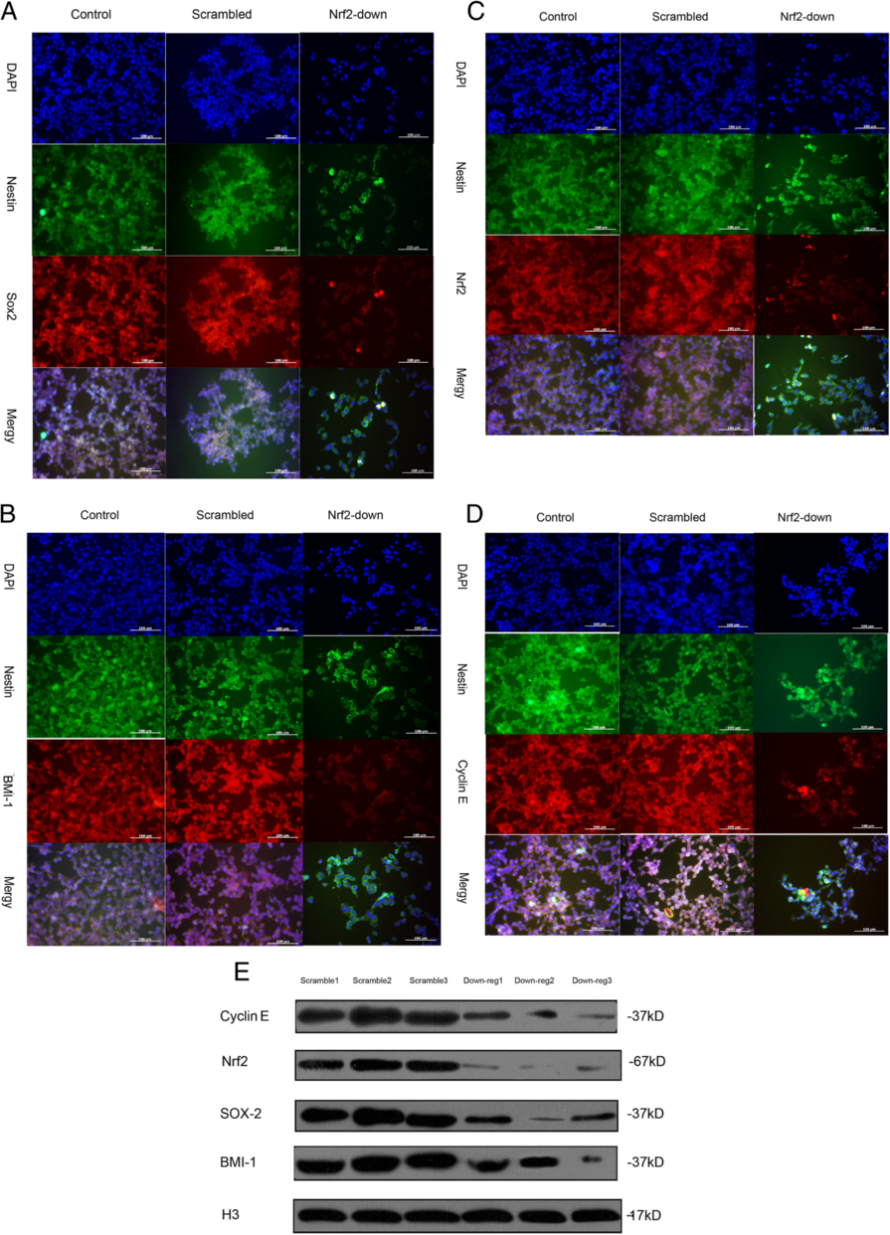
**Immunocytochemistry and western blot assay of Sox2, BMI-1, Nrf2, and CyclinE in GSCs with and without Nrf2 knockdown.** Photomicrographs are arranged from top to bottom as: nuclear staining with DAPI, Nestin and stain for **(A)** Sox2, **(B)** BMI-1, **(C)** Nrf2, and **(D)** CyclinE. They were merged in the bottom of the pictures. The scale bar is representative for all photomicrographs. (**E**) Western blot analyses of nuclear proteins isolated from GSCs. Protein expression levels, presented in parentheses beneath corresponding bands, were normalized against a corresponding H3 loading control (data not shown).

We examined the subcellular localization of Sox2 (Figure 3A), BMI-1 (Figure 3B), Nrf2 (Figure 3C), and cyclin E (Figure 3D). Both the protein BMI-1, Sox2 and Cyclin E is required for maintenance of self-renewal and proliferation [22,23]. Certain kinds of stem cells constitutively active Cdk2–cyclin-E complexes and enhance the proliferation of stem cells. Otherwise, the activity Nrf2 would been dissociated with Keap1 and transported into nuclear [13]. We needed to analysis the markers of self-renewal and the activation of Nrf2. For this purpose, we conducted immunocytochemistry. Specifically, GSCs were infected with either Scrambled or Human Nrf2 shRNA lentiviruses. Following infection, the transduced cells were selected for puromycin resistance for 24 hours, subcultured into 24-well plates, and probed for Nrf2, Sox2, BMI-1 and Cyclin E by immunocytochemistry. Nrf2, Sox2, BMI-1 and Cyclin E proteins were detected within the nucleus of sphere-like cells 72 h after transduction with Scrambled shRNA (Figure 1C and Figure 3E). There was a reduction in the intensity of fluorescence associated with Sox2 (Figure 3A), BMI-1 (Figure 3B), and cyclin E (Figure 3D) after Nrf2 knockdown. Similarly, Nrf2 levels in the nucleus were lower in the down-regulated group. All GSCs highly expressed nestin.

### Nrf2 knockdown in GSCs affect the expression of markers of pluripotency-associated genes

Using qPCR assays, we demonstrated significant decreases in the expression levels of Sox2, BMI-1 (Figure 1B), and cyclin E (Figure 1B). In contrast, there was a small increase in the expression level of GFAP (Figure 1B). We did not observe obvious changes in the expression levels of nestin (Figure 1B). The number of viable cells that expanded was dramatically reduced upon knockdown of Nrf2. This corresponded with our previous observation that Nrf2 knockdown in GSCs reduces their capacity to proliferate and self-renew.

### Nrf2 knockdown enriches the proportion of cells in G2 phase in GSCs

**Figure 4:**
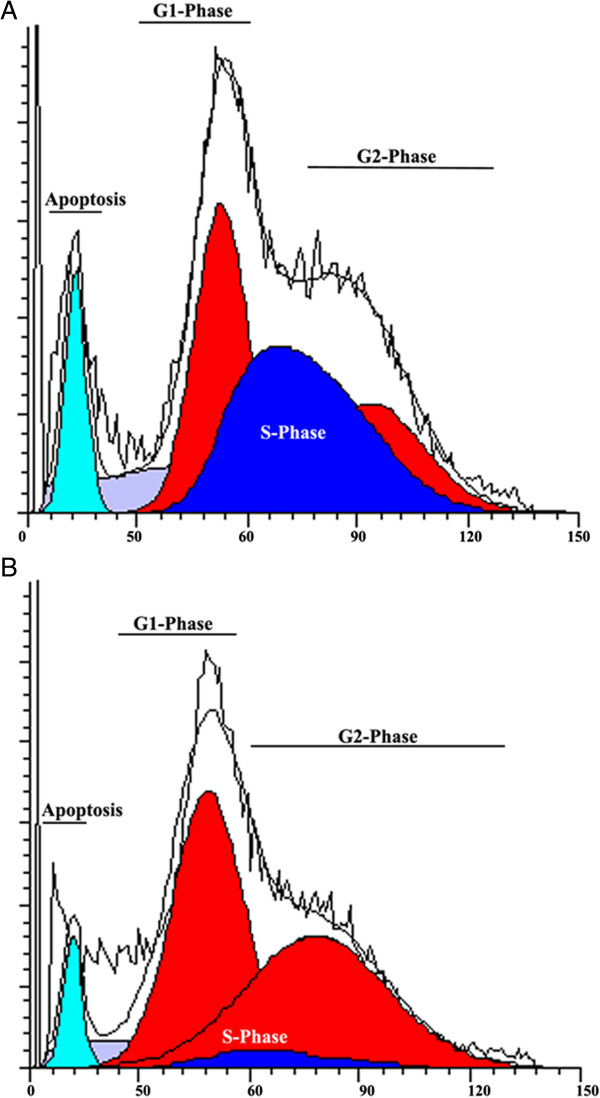
**Effects of cell cycle analysis by propidium iodide (PI) staining and flow cytometry is presented. (A)** The proportion of cells in G2 phase (30.8 ± 2.1%) and S phase (37.13 ± 3.9%) for the Scrambled shRNA. **(B)** The proportion of cells in G2 phase (46.7 ± 4.5%) for the Nrf2 shRNA;(*P* = 0.042). Additionally, there was a corresponding decrease (7.5 ± 0.41%, *P* = 0.0009) in the proportion of cells in S phase.

GSCs were infected with either the Scrambled or Nrf2 shRNA lentivirus and cell cycle analysis was conducted 72 h later (Figure 4). Nrf2 knockdown resulted in a significant increase in the proportion of cells in the G2 phase for the Scrambled (30.8 ± 2.1%) and Nrf2 (46.7 ± 4.5%) shRNAs (*P* = 0.042). Additionally, there was a corresponding decrease (7.5 ± 0.41%, *P* = 0.0009) in the proportion of cells in the S phase (Figure 4).

### Knockdown of Nrf2 attenuates the tumorigenicity of GSCs *in vivo*

**Figure 5:**
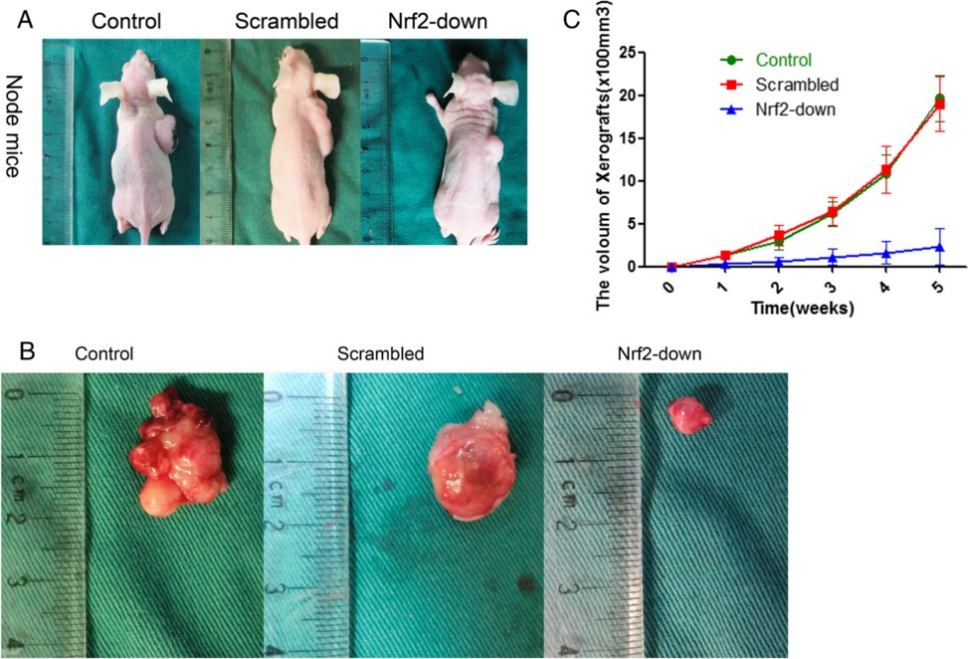
**Knockdown of Nrf2 attenuates the tumorigenicity of GSCs in vivo. (A)** The differences in tumor volume upon harvest of xenograft tumor from the Scrambled or the Nrf2 shRNA lentivirus treated groups in node mices. **(B)** morphology of tumors in the node mices. **(C)** curve of tumor volume in node mices. procreating GSCs treated with Nrf2 shRNA lentivirus resulted in tumor volume of 210 ± 57 mm3, compared with a much larger volume of 1850 ± 260 mm3 in scrambled tumors and 1900 ± 300 mm3 in control tumors.

Animals receiving control GSCs and Scrambled shRNA developed tumors on day 7, while animals receiving treated GSCs did not develop tumors until day 14. Furthermore, there was also a difference in tumor volume upon harvest of xenograft tumor from the Scrambled or the Nrf2 shRNA lentivirus treated groups. As is shown in Figure 5A and Figure 5B, procreating GSCs treated with the Nrf2 shRNA lentivirus resulted in a tumor volume of 210 ± 57 mm^3^, compared with a much larger volume of 1850 ± 260 mm^3^ in tumors treated with the Scrambled shRNA, and control tumors (1900 ± 300 mm^3^; Figure 5A, B, and C).

## Discussion

Recent studies have shown that therapeutic resistance of glioblastomas is due to the presence of viable GSCs that confer tumorigenic potential and a survival advantage against chemotherapy [2,11,24]. This therapeutic resistance is based on many inter- and extra-cell regulatory systems [2,25,26]. These systems allow GSCs to survive under adverse conditions, including hypoxia, nutrient deficiency, radioactive injury, cytotoxicity, and immune system reactions. Previous studies have demonstrated the importance of the anti-hypoxia ability of GSCs [27,28], and predicted the potential of anti-hypoxic cell signaling systems in promoting tumorigenesis of glioblastomas [28-30].

Nrf2, a basic redox-sensitive bZIP transcription factor, is present under anti-hypoxia conditions by influencing the transcription of HO-1 and VEGF. It also activates cytoprotective pathways against oxidative injury, inflammation, and apoptosis via the transcriptional induction of a large number of self-defense genes involved with phase II detoxication enzymes and antioxidant stress enzymes [13,15]. Keap1 negatively regulates Nrf2 activity through ubiquitin-mediated proteasomal degradation. This indicates that complete loss of Keap1 activity leads to constitutive activation of Nrf2 [14,15,31]. High levels of Nrf2 expression in conjunction with temozolomide treatment induce cell autophagy in glioblastomas [17]. In our laboratory, Nrf2 cell signals enhanced the proliferation of U251 and U87 glioblastoma cell lines [18]. Recent studies demonstrated that Nrf2, well established as a global regulator of the oxidative stress response, plays a regulatory role in several kinds of stem cells such as hematopoietic stem cells and NSCs [32-35]. Nrf2 regulates hematopoietic stem cell survival, but this process may not be dependent upon reactive oxidative species (ROS). These results also suggest that the NRF2/antioxidant response element signaling pathway has the potential to induce fetal hemoglobin, indicating that Nrf2 plays a critical role in stem cells. Taken together, these lines of evidence demonstrate the important role of GSCs during self-renewal and during glioblastoma relapses, and the observations support the idea that a reduction in the Nrf2-dependent protective response may down-regulate the self-renewal of GSCs.

Using RNA interference (RNAi) technology, Nrf2 was knocked down in the GSCs of three patients, and GSC cloning efficiency was significantly decreased when Nrf2 was knocked down. The *BMI-1* gene is a polycomb gene family transcriptional repressor and a proto-oncogene. The protein BMI-1 is required for maintaining self-renewal and proliferation [36]. Sox2 is a member of the *Sox* gene family and has been shown to be related to the sex-determining gene *Sry* and a regulator of the *FGF-4* gene, which is essential for the self-renewal and pluripotency of NSCs [22,23]. Some stem cells constitutively express Cdk2–cyclin-E complexes and enhance the proliferation of stem cells. Cyclin E binds to G_1_ phase Cdk2, which is required for the transition from the G_1_ to the S phase of the cell cycle that determines cell division. In our study, knocking down of Nrf2 in GSCs leads to decreased expression levels of pluripotency-associated transcription factors such as BMI-1, Sox2 and cyclin E, and an increase in the expression of markers associated with astrocyte development.

In our work we showed that transient exposure of GSCs to Nrf2 shRNAs *ex vivo* was capable of inhibiting tumorigenicity in nude mice. We inferred that the Nrf2 pathway is indispensable for the self-renewal of GSCs both *in vivo* and *in vitro*. Knocking down Nrf2 expression reduced the capacity of self-renewal and tumorigenesis *in vivo*. Nrf2 may be a potential target for controlling the growth of glioblastomas in patients.

Our studies also demonstrate a significant enrichment in the proportion of cells in the G2–M phase of the cell cycle. We also observed a significant decrease in S-phase cells when Nrf2 was knocked down in GSCs. The cell cycle of most somatic cells is regulated by the G1 checkpoint that restricts the G1–S transition until the formation of activated cyclin-dependent kinases [37]. Certain stem cells lack a G1 restriction point because of a constitutively active Cdk2–cyclin-E complex. Both stem cells and somatic cells possess a checkpoint between the G2 and M phases of the cell cycle. In the case of GSCs, cyclin E levels oscillate; when active Cdk2–cyclin E complexes form, the cells are able to enter M phase. Therefore it is not a surprise that cell cycle defects in GSCs lead to an accumulation of cells in the G2–M phases.

It is conceivable that Nrf2 plays more than one essential role in GSCs. To date, a diverse set of biological functions have been described for Nrf2. It is a key nuclear transcription factor that regulates ARE-containing genes [13,31]. Knocking down Nrf2 expression decreases the self-renewing activity of GSCs, thereby suggesting that it plays an important role in regulating GSC proliferation. Recent studies suggest that many factors, including Nrf2, influence the self-renewal of stem cells, the cyto-construction system [38,39], the cell cycle and check point-related proteins, transcriptional factors [36], cell growth factors [40,41], adhesion molecules [42,43], chemotactic factors [44,45], and inter-cell signal transduction pathways [46,47]. In general, Nrf2 is transferred into the nucleus and binds to certain regions of DNA in a sequence-independent manner [13]. These regions can contain the promoter of self-renewal-related molecules [13,14]. Reactive oxygen species (ROS) act as intracellular signaling molecules during anti-oxygen processes. Cellular protective mechanisms against oxidative stress include transcriptional control of cytoprotective enzymes by Nrf2. [48]. In our study, we chose BMI-1, Sox2 and Cyclin E as candidates for determining the possible mechanisms of Nrf2 during GSC self-renewal. BMI-1 is an important transcription regulatory factor in stem cells. Sox2 regulates the secretion of many growth factors such as FGF and Oct4. Cyclin E is a check point protein, which restricts the cell cycle in GSCs. Both western blotting and qPCR assays verified the decrease of these factors through Nrf2 knockdown.

In this study we did not explore the molecular mechanisms of Nrf2 in regulating the self-renewal of GSCs. We did not discuss the cross-reactivity of Nrf2, BMI-1, Sox2 and cyclin E, or even their relationship to ROS. In a further study, we will attempt to explore the relationships between these factors and elucidate the mechanisms of the Nrf2 cell signaling pathway in regulating self-renewal.

In conclusion, we have demonstrated that Nrf2 contributes to maintaining self-renewal in GSCs. Identification of additional proteins that associate with master regulators of the GSC fate will continue to reveal crucial mechanisms and machinery driving the fundamental process of self-renewal. Future efforts to develop cell-based therapies for glioblastomas will no doubt benefit from advances in the basic understanding of the molecular machinery that controls the fate of GSCs.

## Conclusions

Nrf2 is required to maintain the self-renewal of GSCs. Down regulating of Nrf2 by lentivirus can attenuate the self-renewal of GSCs significantly.

## Abbreviations

GSCs: Glioma stem cells; Nrf2: Nuclear factor rythroid 2-related factor 2; ARE: Antioxidant response element.

## Competing interests

The authors declare that they have no competing interests.

## Authors’ contributions

JZ carried out the design of experiment, cell culture, lentivirus, western blot and immunoassays and drafted the manuscript. HW assigned the research plan and prepared the lab meeting group. QS carried out the real-time PCR. XJ participated in the flow cytometry. LZ participated in the design of the study and performed the statistical analysis. ZC helped to draft the manuscript. YZ fetched the tissues from operation. HL helped to plant the cells to the nude mice. MZ helped to alter the manuscript. All authors read and approved the final manuscript.

## Pre-publication history

The pre-publication history for this paper can be accessed here:

http://www.biomedcentral.com/1471-2407/13/380/prepub
