# Correction to: Nrf2 is required to maintain the self-renewal of glioma stem cells

**DOI:** 10.1186/s12885-021-08338-x

**Published:** 2021-05-21

**Authors:** Jianhong Zhu, Handong Wang, Qing Sun, Xiangjun Ji, Lin Zhu, Zixiang Cong, Yuan Zhou, Huandong Liu, Mengliang Zhou

**Affiliations:** 1 Medical School of Nanjing University, No. 22, Hankou Road, Nanjing, 210089 Jiangsu China; 2 Department of Neurosurgery in Jinling Hospital, Neurosurgical Institution of Peoples Liberation Army of China, No. 305, East Zhongshan Road, Nanjing, 210002 Jiangsu China; 3 Neurosurgery Department of Southern Medical University, No. 1838, Guangzhou Avenue, Guangzhou, 510515 Guangdong China

**Correction to: BMC Cancer 13, 380 (2013)**

**https://doi.org/10.1186/1471-2407-13-380**

Following publication of the original article [1], the authors reported the following errors and omissions:

- Figure 1A: In the published article two panels in Fig. 1A are identical (Scrambled/patient 1 and Control/Patient 3). The correct images are presented here.
- Figure 3E: The first four lanes of Cyclin E and SOX-2 are very similar and appear as the same samples at different exposures. The correct images are presented here.
- Figure 3E and 1C present the very same Nrf2 and H3 data. The authors explained they used the same Nrf2 and H3 data because both figures showed the change of Nrf2 expression after downregulation of Nrf2 with lentivirus. This was not made clear in the original figure legends.

None of these changes impair the study outcome or its interpretation in any way.

**Figure 1A**

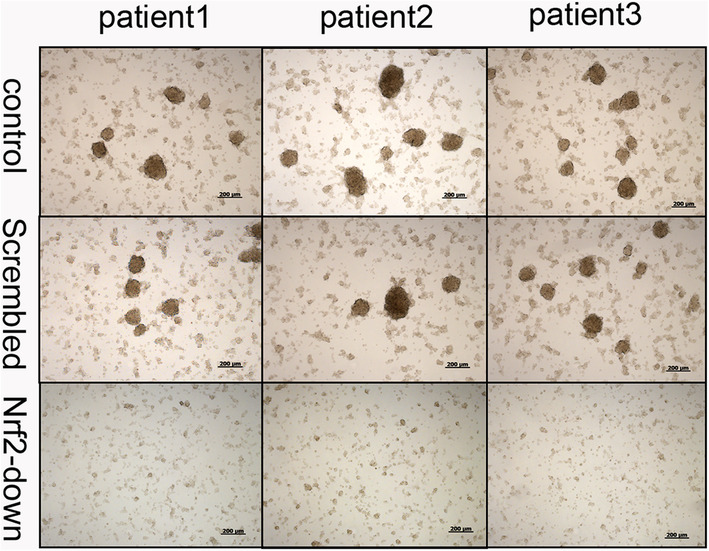



**Figure 3E**


